# Normal and pathogenic variation of *RFC1* repeat expansions: implications for clinical diagnosis

**DOI:** 10.1093/brain/awad240

**Published:** 2023-07-14

**Authors:** Natalia Dominik, Stefania Magri, Riccardo Currò, Elena Abati, Stefano Facchini, Marinella Corbetta, Hannah Macpherson, Daniela Di Bella, Elisa Sarto, Igor Stevanovski, Sanjog R Chintalaphani, Fulya Akcimen, Arianna Manini, Elisa Vegezzi, Ilaria Quartesan, Kylie-Ann Montgomery, Valentina Pirota, Emmanuele Crespan, Cecilia Perini, Glenda Paola Grupelli, Pedro J Tomaselli, Wilson Marques, Genomics England Research Consortium, J C Ambrose, P Arumugam, E L Baple, M Bleda, F Boardman-Pretty, J M Boissiere, C R Boustred, H Brittain, M J Caulfield, G C Chan, C E H Craig, L C Daugherty, A de Burca, A Devereau, G Elgar, R E Foulger, T Fowler, P Furió-Tarí, E Gustavsson, J M Hackett, D Halai, A Hamblin, S Henderson, J E Holman, T J P Hubbard, K Ibáñez, R Jackson, L J Jones, D Kasperaviciute, M Kayikci, L Lahnstein, K Lawson, S E A Leigh, I U S Leong, F J Lopez, F Maleady-Crowe, J Mason, E M McDonagh, L Moutsianas, M Mueller, N Murugaesu, A C Need, C A Odhams, C Patch, D Perez-Gil, D Polychronopoulos, J Pullinger, T Rahim, A Rendon, P Riesgo-Ferreiro, T Rogers, M Ryten, B Rugginini, K Savage, K Sawant, R H Scott, A Siddiq, A Sieghart, D Smedley, K R Smith, A Sosinsky, W Spooner, H E Stevens, A Stuckey, R Sultana, E R A Thomas, S R Thompson, C Tregidgo, A Tucci, E Walsh, S A Watters, M J Welland, E Williams, K Witkowska, S M Wood, M Zarowiecki, Joseph Shaw, James Polke, Ettore Salsano, Silvia Fenu, Davide Pareyson, Chiara Pisciotta, George K Tofaris, Andrea H Nemeth, John Ealing, Aleksandar Radunovic, Seamus Kearney, Kishore R Kumar, Steve Vucic, Marina Kennerson, Mary M Reilly, Henry Houlden, Ira Deveson, Arianna Tucci, Franco Taroni, Andrea Cortese

**Affiliations:** Department of Neuromuscular Diseases, University College London, London WC1N 3BG, UK; Unit of Medical Genetics and Neurogenetics, Fondazione IRCCS Istituto Neurologico Carlo Besta, Milan 20133, Italy; Department of Neuromuscular Diseases, University College London, London WC1N 3BG, UK; Department of Brain and Behavioral Sciences, University of Pavia, Pavia 27100, Italy; Department of Neuromuscular Diseases, University College London, London WC1N 3BG, UK; Department of Pathophysiology and Transplantation, University of Milan, Milan 20122, Italy; Department of Neuromuscular Diseases, University College London, London WC1N 3BG, UK; IRCCS Mondino Foundation, Pavia 27100, Italy; Unit of Medical Genetics and Neurogenetics, Fondazione IRCCS Istituto Neurologico Carlo Besta, Milan 20133, Italy; Department of Neuromuscular Diseases, University College London, London WC1N 3BG, UK; Unit of Medical Genetics and Neurogenetics, Fondazione IRCCS Istituto Neurologico Carlo Besta, Milan 20133, Italy; Unit of Medical Genetics and Neurogenetics, Fondazione IRCCS Istituto Neurologico Carlo Besta, Milan 20133, Italy; Genomics Pillar, Garvan Institute of Medical Research, Sydney 2010, Australia; Centre for Population Genomics, Garvan Institute of Medical Research and Murdoch Children’s Research Institute, Darlinghurst 2010, Australia; Centre for Population Genomics, Garvan Institute of Medical Research and Murdoch Children’s Research Institute, Darlinghurst 2010, Australia; Laboratory of Neurogenetics, National Institute on Aging, National Institutes of Health, Bethesda, MD 2292, USA; Department of Neuromuscular Diseases, University College London, London WC1N 3BG, UK; Department of Pathophysiology and Transplantation, University of Milan, Milan 20122, Italy; Department of Neurology and Laboratory of Neuroscience, IRCCS Istituto Auxologico Italiano, Milan 20145, Italy; IRCCS Mondino Foundation, Pavia 27100, Italy; Department of Brain and Behavioral Sciences, University of Pavia, Pavia 27100, Italy; Department of Neuromuscular Diseases, University College London, London WC1N 3BG, UK; Department of Chemistry, University of Pavia, Pavia 27100, Italy; G4-INTERACT, USERN, 27100 Pavia, Italy; Institute of Molecular Genetics IGM-CNR ‘Luigi Luca Cavalli-Sforza’, Pavia 27100, Italy; Institute of Molecular Genetics IGM-CNR ‘Luigi Luca Cavalli-Sforza’, Pavia 27100, Italy; Institute of Molecular Genetics IGM-CNR ‘Luigi Luca Cavalli-Sforza’, Pavia 27100, Italy; Department of Neurology, School of Medicine of Ribeirão Preto, University of São Paulo, Ribeirão Preto 2650, Brazil; Department of Neurology, School of Medicine of Ribeirão Preto, University of São Paulo, Ribeirão Preto 2650, Brazil; Department of Neuromuscular Diseases, University College London, London WC1N 3BG, UK; Department of Neuromuscular Diseases, University College London, London WC1N 3BG, UK; Clinic of Central and Peripheral Degenerative Neuropathies Unit, IRCCS Foundation, C. Besta Neurological Institute, Milan 20126, Italy; Clinic of Central and Peripheral Degenerative Neuropathies Unit, IRCCS Foundation, C. Besta Neurological Institute, Milan 20126, Italy; Clinic of Central and Peripheral Degenerative Neuropathies Unit, IRCCS Foundation, C. Besta Neurological Institute, Milan 20126, Italy; Clinic of Central and Peripheral Degenerative Neuropathies Unit, IRCCS Foundation, C. Besta Neurological Institute, Milan 20126, Italy; Nuffield Department of Clinical Neurosciences, University of Oxford, Oxford OX3 9DU, UK; Nuffield Department of Clinical Neurosciences, University of Oxford, Oxford OX3 9DU, UK; Oxford Centre for Genomic Medicine, Oxford University Hospitals NHS Foundation Trust, Oxford OX3 7HE, UK; Salford Royal NHS Foundation Trust Greater Manchester Neuroscience Centre, Manchester Centre for Clinical Neurosciences Salford, Greater Manchester M6 8HD, UK; Barts MND Centre, Royal London Hospital, London E1 1BB, UK; Department of Neurology, Royal Victoria Hospital, Belfast BT12 6BA, UK; Kinghorn Centre for Clinical Genomics, Garvan Institute of Medical Research, Darlinghurst, NSW 2010, Australia; Molecular Medicine Laboratory, Concord Hospital, Concord, NSW 2139, Australia; Concord Clinical School, Faculty of Medicine and Health, University of Sydney, Sydney, NSW 2139, Australia; Concord Clinical School, Faculty of Medicine and Health, University of Sydney, Sydney, NSW 2139, Australia; Brain and Nerve Research Centre, Concord Hospital, Sydney, NSW 2139, Australia; Molecular Medicine Laboratory, Concord Hospital, Concord, NSW 2139, Australia; Northcott Neuroscience Laboratory, ANZAC Research Institute SLHD, Sydney, NSW 2050, Australia; School of Medical Sciences, Faculty of Medicine and Health, University of Sydney, Sydney, NSW 2050, Australia; Department of Neuromuscular Diseases, University College London, London WC1N 3BG, UK; Department of Neuromuscular Diseases, University College London, London WC1N 3BG, UK; Genomics Pillar, Garvan Institute of Medical Research, Sydney 2010, Australia; Department of Neuromuscular Diseases, University College London, London WC1N 3BG, UK; Unit of Medical Genetics and Neurogenetics, Fondazione IRCCS Istituto Neurologico Carlo Besta, Milan 20133, Italy; Department of Neuromuscular Diseases, University College London, London WC1N 3BG, UK; Department of Brain and Behavioral Sciences, University of Pavia, Pavia 27100, Italy

**Keywords:** *RFC1*, CANVAS, ataxia, neuropathy, repeat expansions, long-read sequencing

## Abstract

Cerebellar ataxia, neuropathy and vestibular areflexia syndrome (CANVAS) is an autosomal recessive neurodegenerative disease, usually caused by biallelic AAGGG repeat expansions in *RFC1*. In this study, we leveraged whole genome sequencing data from nearly 10 000 individuals recruited within the Genomics England sequencing project to investigate the normal and pathogenic variation of the *RFC1* repeat. We identified three novel repeat motifs, AGGGC (*n* = 6 from five families), AAGGC (*n* = 2 from one family) and AGAGG (*n* = 1), associated with CANVAS in the homozygous or compound heterozygous state with the common pathogenic AAGGG expansion. While AAAAG, AAAGGG and AAGAG expansions appear to be benign, we revealed a pathogenic role for large AAAGG repeat configuration expansions (*n* = 5). Long-read sequencing was used to characterize the entire repeat sequence, and six patients exhibited a pure AGGGC expansion, while the other patients presented complex motifs with AAGGG or AAAGG interruptions. All pathogenic motifs appeared to have arisen from a common haplotype and were predicted to form highly stable G quadruplexes, which have previously been demonstrated to affect gene transcription in other conditions.

The assessment of these novel configurations is warranted in CANVAS patients with negative or inconclusive genetic testing. Particular attention should be paid to carriers of compound AAGGG/AAAGG expansions when the AAAGG motif is very large (>500 repeats) or the AAGGG motif is interrupted. Accurate sizing and full sequencing of the satellite repeat with long-read sequencing is recommended in clinically selected cases to enable accurate molecular diagnosis and counsel patients and their families.

## Teaser

*RFC1* expansions are a common cause of ataxia and sensory neuropathy. Dominik *et al.* investigate normal and pathogenic variation of the *RFC1* repeat and identify three novel repeat configurations associated with the CANVAS disease spectrum. The size and GC content of repeats may be more important than the exact repeat motif.

## Introduction

Cerebellar ataxia, neuropathy and vestibular areflexia syndrome (CANVAS) is an autosomal recessive neurodegenerative disease characterized by adult onset and slowly progressive ataxia caused by the concurrent impairment of sensory neurons, the vestibular system and the cerebellum. In most cases, the disease is caused by biallelic AAGGG repeat expansions in the second intron of the replication factor complex subunit 1 (*RFC1*) gene.^1-19^ Additional pathogenic (AAAGG)_10–25_(AAGGG)*_n_* and ACAGG configurations have been identified in people from Oceania and East Asia, suggesting the possibility that genetic heterogeneity at the repeat locus underlies this condition.^20-23^

In this study, we leveraged whole genome sequencing (WGS) data from the 100,000 Genomes Project to investigate the normal and pathogenic variations of the *RFC1* repeat and identify additional pathogenic motifs that cause CANVAS. These were further analysed using targeted long-read sequencing.

We identified three novel pathogenic repeat configurations, AAGGC, AGGGC and AGAGG, either in the homozygous or compound heterozygous state with AAGGG repeats, which were similar or larger in size compared with the common AAGGG expansion. In addition, pathogenic uninterrupted or interrupted AAAGG expansions were identified, which were significantly larger in size than the more frequent non-pathogenic AAAGG repeat.

## Materials and methods

### Whole genome sequencing data analysis

The 100,000 Genomes Project, run by Genomics England (GEL), was established to sequence whole genomes of UK National Health Service (NHS) patients affected by rare diseases and cancer.^24^ In this study, we leveraged GEL WGS data and screened for the presence of pentanucleotide expansions in *RFC1* in 893 samples from patients diagnosed with ataxia and 8107 controls, all aged 30 years or older. Repeat expansions were detected using ExpansionHunterDeNovo (EHDN) v0.9.0. We considered all motifs composed of five or six nucleotides at the *RFC1* locus. Repeat motifs present in the homozygous or compound heterozygous state with the AAGGG expansion in ataxia cases, but absent or significantly less frequent in controls, were considered to be possibly pathogenic and were further assessed.

Structural variants were detected using Manta^25^ as described at https://re-docs.genomicsengland.co.uk/genomic_data/.

Predicted genetic ancestries for samples from GEL were based on a principal component analysis (PCA), using the five macro-ethnicities of the 1000 Genomes project (European, African, South Asian, East Asian, American) as reference populations. Samples in which none of the components reached 95% were classified as ‘Mixed’.

### Repeat-primed-PCR

Samples identified to carry novel pathogenic repeat motifs with EHDN were tested using repeat-primed (RP)-PCR. In addition, we screened a cohort of 540 samples with genetically confirmed *RFC1* CANVAS, as defined by the presence of a positive RP-PCR for the AAGGG expansion and the absence of an amplifiable PCR product from the flanking PCR, to look for expansions of different repeat motifs on the second allele. RP-PCR for AAAAG, AAAGG and AAGGG expansions was performed as previously described.^1^ The following primers were used: AGGGC-Rv: 5′-CAGGAAACAGCTATGACCAACAGAGCAAGACTCTGTTTCAAAAAGGGCAGGGCAGGGCAGGGCA-3′; AAGGC-Rv; 5′-AAGGC: CAGGAAACAGCTATGACCAACAGAGCAAGACTCTGTTTCAAAAAGGCAAGGCAAGGCAA-3′; or AGAGG-Rv: 5′-CAGGAAACAGCTATGACCAACAGAGCAAGACTCTGTTTCAAAAAGGAGAGGAGAGGAGAGGAGA-3′, depending on the configuration tested. The PCR conditions for AGGGC and AAGGC were modified to 30 s denaturation per cycle as opposed to 10 s for all other configurations.

### Southern blotting

Briefly, 5 µg of high molecular weight (HMW) DNA was enzymatically digested with EcoRI for 3 h and size-fractionated on a 1.2% agarose gel for 15 h. The gel was washed in depurination, denaturing and neutralizing solutions for 45 min each, after which the blot was assembled to transfer DNA from the gel onto a positively-charged membrane using an upward transfer method for 15 h. The DNA was UV-crosslinked to the membrane and hybridized with a mixture of salmon sperm and *RFC1* probe in digoxigenin granules (DIG) solution (Roche) overnight. The membrane was then washed, blocked and anti-DIG antibody was added, after which detection buffer and CDP-STAR chemiluminescent substrate (Roche) were used to visualize hybridization fragments.

### Targeted *RFC1* long-read sequencing

We performed long-read sequencing to establish the precise repeat sequence in patients carrying a novel, likely pathogenic, expansion of *RFC1*. Given the technical hurdle of sequencing large repeat expansions, samples were sequenced on different platforms, including those from Oxford Nanopore and Pacific Biosciences (PacBio). Target enrichment was performed with either a clustered regularly interspaced short palindromic repeats (CRISPR)-associated protein-9 nuclease (Cas9) system or ReadUntil programmable selective sequencing.

Samples were extracted from blood using the Qiagen MagAttract HMW DNA kit and quality was checked using readouts from a Thermo Scientific NanoDrop system. For CRISPR/Cas9-targeted sequencing, fragment lengths were assessed using the Agilent Femto Pulse Genomic DNA 165 kb kit, and only samples in which the majority of the fragments were over 25 kb were used. Libraries were prepared from 5 µg of input DNA for each sample for both the PacBio No-Amp targeted sequencing utilizing the CRISPR-Cas9 system protocol (Version 09) and the Oxford Nanopore ligation sequencing gDNA Cas9 enrichment (SQK-LSK109) protocol (Version: ENR_9084_v109_revT_04Dec2018). Libraries were sequenced on the Oxford Nanopore PromethION or MinION platforms or the PacBio Sequel IIe, respectively. For the Oxford Nanopore ligation sequencing gDNA Cas9 enrichment, we used four CRISPR-Cas9 guides from Nakamura *et al*.,^22^ RFC1-F1: 5′-GACAGTAACTGTACCACAATGGG-3′, RFC1-R1: 5′-CTATATTCGTGGAACTATCTTGG-3′, RFC1-F2: 5′-ACACTCTTTGAAGGAATAACAGG-3′ and RFC1-R2: 5′-TGAGGTATGAATCATCCTGAGGG-3′, except for Cases IV-1, XI-1 and XII-1, for which only two, RFC1-F2 and RFC1-R2, were used. The guides RFC1-F3: 5′-GAAACTAAATAGAACCAGCC-3′ and RFC1-R3: 5′-GACTATGGCTTACCTGAGTG-3′, designed in-house, were used for PacBio No-Amp targeted sequencing, and up to 10 samples were multiplexed using PacBio barcoded adapters. Libraries loaded onto the PromethION and MinION were run for 72 h with standard loading protocols. Sequel IIe libraries were run for a movie time of 30 h with an immobilization time of 4 h. All libraries were loaded neat.

Programmable targeted sequencing was performed as described previously.^26^ HMW DNA was sheared to fragment sizes of ∼20 kb using Covaris G-tubes. Sequencing libraries were prepared from ∼3–5 μg of HMW DNA using a native library prep kit SQK-LSK110, according to the manufacturer’s instructions. Each library was loaded onto a FLO-MIN106D (R9.4.1) flow cell and run on an ONT MinION device with live target selection/rejection executed by the ReadFish software package.^27^ Detailed descriptions of the software and hardware configurations used for the ReadFish experiments are provided in a recent publication that demonstrates the suitability of this approach for profiling tandem repeats.^26^ The target used in this study was the *RFC1* gene locus ±50 kb. Samples were run for a maximum duration of 72 h, with nuclease flushes and library reloading performed at approximately 24 and 48 h time-points for targeted sequencing runs, to maximize sequencing yield.

### Bioinformatic analysis

Alignment to the hg38 reference of Nanopore reads, PacBio CCS and PacBio subreads was done using minimap2^28^ with additional options ‘-r 10000 -g 20000 -E 4,0’. For PacBio sequences, the recommended step of generating circular consensus sequencing (CCS) maps from subreads was not always possible because of the low depth of the sequencing data. The only CCS map we could obtain was for the AAGGG allele in Case V-1. After alignment, we used PacBio scripts (https://github.com/PacificBiosciences/apps-scripts) to extract the repeat region (extractRegions.py) and obtain waterfall plots (waterfall.py) for the following motifs: AAGGG, AGAGG, AGGGC, AAGGC and AAAGG.

For programmable targeted sequencing, raw ONT sequencing data were converted to BLOW5 format using slow5tools (v0.3.0)^29^ then base-called using Guppy (v6). The resulting FASTQ files were aligned to the hg38 reference genome using minimap2 (v2.14-r883). The short-tandem repeat (STR) site within the *RFC1* locus was genotyped using a process validated in our recent manuscript.^27^ This method involves the local haplotype-aware assembly of ONT reads spanning a given STR site and annotation of the STR size, motif and other summary statistics using Tandem Repeats Finder (4.09), followed by manual inspection and motif counting.

### Haplotype analysis

We used SHAPEITv4^30^ with default parameters to phase a 2 Mb region (chr4:38020000–40550000) encompassing the *RFC1* gene. To maximize available haplotype information, the entire Rare Diseases panel in Genomics England (78 195 samples from patients affected by rare diseases) were jointly phased. The input data format was an aggregate VCF file with a total of 551 795 variants.

The estimation of haplotype age was based on the online application Genetic Mutation Age Estimator (https://shiny.wehi.edu.au/rafehi.h/mutation-dating/).^31^ The method required as input a list of ancestral segments for sampled individuals. We used the five individuals with pathogenic expansions (Fig. 3): AAGGG hom, ACAGG hom, Case VII-1, Case I-1 and Case III-3.

### Optical genome mapping

Patients for whom whole blood was available were subjected to BioNano optical genome mapping (OGM) to gather additional information on the precise size of the expanded repeat. Ultra HMW genomic DNA was isolated as described by the Bionano prep SP frozen human blood DNA isolation protocol v2. Homogeneous ultra HMW DNA was labelled using the Bionano prep direct label and stain (DLS) protocol provided with the kit, and the homogeneous labelled DNA was loaded onto a Saphyr chip. Optical mapping was performed at a theoretical coverage of 400×. Molecule files (.bnx) were aligned to hg38 with Bionano Solve script ‘align_bnx_to_cmap.py’ from Bionano Solve v3.6 (https://bionano.com/software-downloads/) using standard parameters. For each sample, molecules overlapping both markers flanking the repeat expansion were extracted (marker IDs: 7723 and 7724). Intermarker distances were analysed by decomposing into two Gaussian components, and using the Gaussian mean as the allele size, the repeat expansion size was calculated as the difference between the Gaussian mean and the intermarker distance of a non-expanded allele (6858 bp).

### G-quadruplexes

The propensity of the different repeat configurations in *RFC1* to form G-quadruplexes (G4s)^32^ was predicted using the Quadruplex forming G-Rich Sequences (QGRS) Mapper^33^ and G4-Hunter software,^34^ through which the likelihood to form a stable G4 is rated in terms of G-score values. Putative G4s were identified according to the following parameters for QGRS: a maximum sequence length of 30 nucleotides, minimum number of two G-tetrads in a G4, loop lengths in the range of 0–36 nucleotides and G-score values > 15. The G4-Hunter threshold was 1.5 with a window size of 20 nucleotides.

## Results

### Novel pathogenic repeat motifs in *RFC1* in patients from the 100,000 Genome project

Of 893 cases diagnosed with adult-onset ataxia (over the age of 30 years) recruited as part of the 100,000 Genome project, 124 cases harboured at least one AAGGG repeat expansion and 48 had biallelic AAGGG repeat expansions, thus confirming a diagnosis of CANVAS/spectrum disorder.

To identify additional likely pathogenic repeat motifs in *RFC1*, we specifically looked for rare repeat configurations present in patients diagnosed with adult-onset ataxia (over the age of 30 years) or in a compound heterozygous state with the known pathogenic AAGGG repeat expansion but absent or significantly less frequent in controls under the same conditions (Table 1).

**Table 1:** Normal and pathogenic variations of the *RFC1* repeat locus in patients from the 100,000 Genome Project

	Hereditary ataxia (*n* = 893)	Non-neurological controls (*n* = 8107)	*P*-values
**Rare homozygous (<1%) repeat expansions present in ataxia cases and absent in controls**			
ACAGG (hom)	1 (0.01%)	0 (0%)	–
**AAGGC (hom)**	1 (0.01%)	0 (0%)	–
**Repeat expansion found in compound heterozygous state with AAGGG expansions (allele 1/allele 2)**			
AAGGG/AAAAG	21 (2.3%)	248 (3%)	ns
AAGGG/AAAGGG	5 (0.6%)	32 (0.4%)	ns
AAGGG/AAGAG	3 (0.3%)	16 (0.2%)	ns
AAGGG/**AAAGG**	10 (1.1%)	47 (0.6%)	0.05
AAGGG/ACGGG^a^	1 (0.01%)	0 (0%)	–
AAGGG/**AGAGG**	1 (0.01%)	0 (0%)	–
AAGGG/**AGGGC**	1 (0.01%)	0 (0%)	–

Novel pathogenic repeat motifs identified in this study are highlighted in bold. hom = homozygous; ns = not significant.

^a^Small (ACGGG)_50_ expansion in the typical non-pathogenic range (10–220).

We identified three cases carrying repeat expansions AAGGC (Case I-1), AGGGC (Case II-1) or AGAGG (Case VII-1) repeat motifs, which were absent in non-neurological controls. AAGGC was present in the homozygous state, while AGGGC and AGAGG were in the compound heterozygous state with the AAGGG expansion. One additional case with self-reported Asian ancestry carried the previously reported rare pathogenic ACAGG repeat expansion in the homozygous state.

AAAAG, AAAGGG and AAGAG expansions were found at similar frequencies in patients and controls, supporting their non-pathogenic significance, while there was a higher percentage of compound heterozygous AAGGG/AAAGG carriers in ataxia cases (*P* = 0.05).

All predicted genetic ancestries for individuals carrying rare homozygous or compound heterozygous expansions in *RFC1* are reported in Supplementary Table 2. Patients carrying AAGGC (Case I-1) and AGGGC (Case II-1) expansions were of predicted South Asian and mixed ethnicity, respectively; an ACAGG expansion carrier was confirmed to be East Asian based on the predicted genetic ancestry, while other repeat configurations were mostly identified in individuals of European or mixed ethnicity.

We did not identify any loss-of-function variant or structural variant in the *RFC1* gene in individuals carrying heterozygous AAGGG repeat expansions.

**Figure 1:**
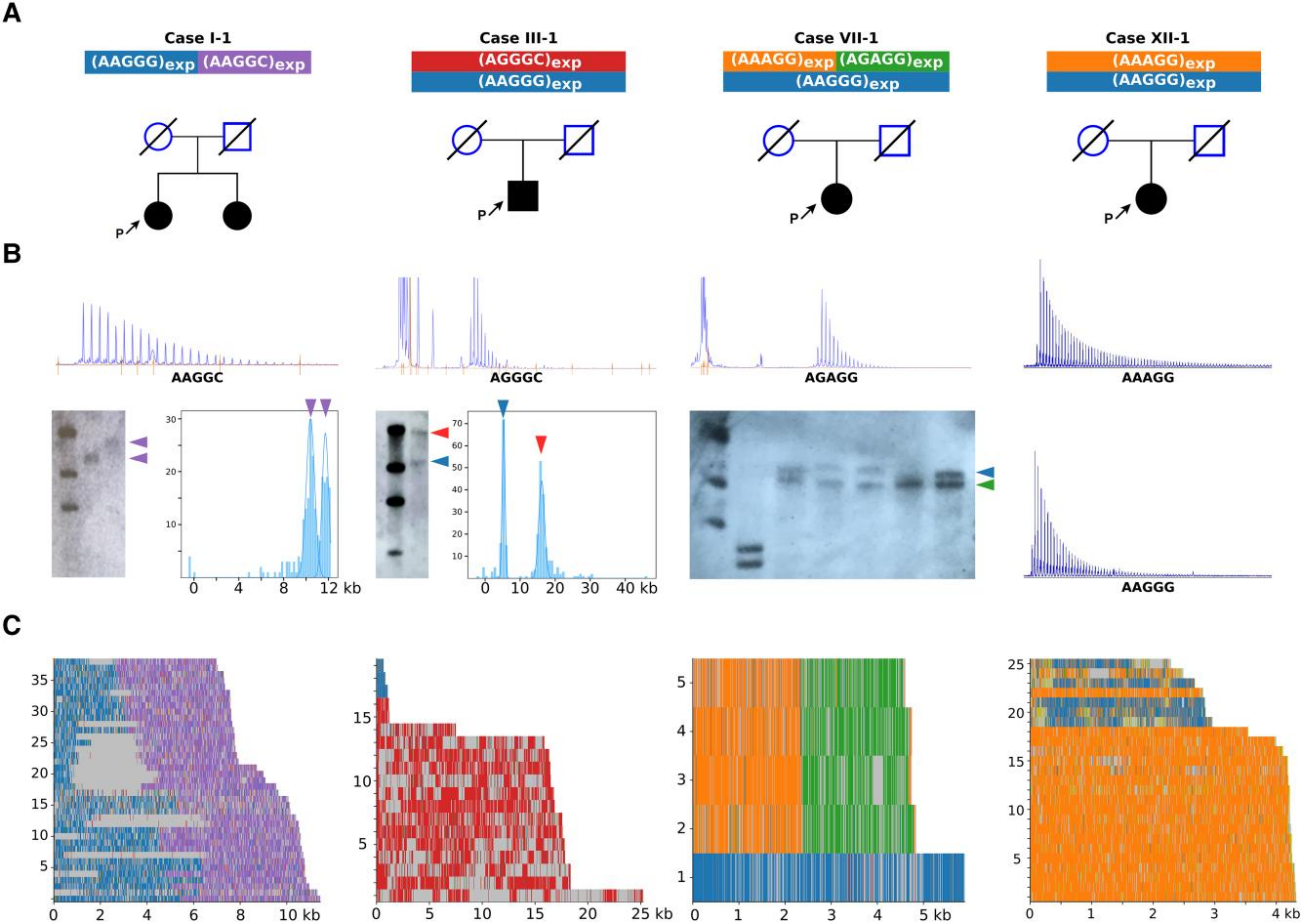
**Long-read sequencing defines the precise sequence of the novel pathogenic *RFC1* motifs.** (**A**) Pedigrees. P = proband. (**B**) RP-PCR plots and, where available, Southern blot images and optical genome mapping plots. (**C**) Long-read sequencing results of representative patients with AAGGC, AGGGC, AGAGG and AAAGG expansions (Cases I-1, III-1, VII-1 and XII-1). In Case III-1, only partial reads, which did not span the entire *RFC1* repeat locus, could be obtained from the AAGGG allele.

The presence of AGGGC, AAGGC or AGAGG repeat expansions was confirmed by RP-PCR in all three cases, and the AAGGC repeat segregated with the disease in Family I, as it was also present in the affected sister Case I-2 (Fig. 1A). Additionally, one case with isolated cerebellar ataxia carried the AAGGG expansion along with an ACGGG repeat, which was absent in the controls. However, Sanger sequencing showed that the ACGGG expansion was only 50 repeats, which is considerably below the lower limit of pathogenicity (250 repeats) for the pathogenic AAGGG motifs and was therefore considered likely to be non-pathogenic in this case. Notably, the patient exhibited isolated cerebellar ataxia but no neuropathy, which is unusual in *RFC1* disease.

Next, we used RP-PCR to screen an internal cohort of 540 DNA samples from cases with sensory neuropathy, ataxia or CANVAS and identified five additional cases carrying an AGGGC expansion (Cases III-1, IV-1, V-1, V-2 and VI-1) and three cases carrying AAAGG expansions on the second allele (Cases X-1, XI-1 and XII-1) (Table 2). We did not identify additional AGAGG or AAGGC repeat expansion carriers. All cases were of self-reported Caucasian ethnicity.

**Table 2:** Clinical and demographic features of patients carrying novel pathogenic repeat configurations in *RFC1*

	*RFC1* genotype	Sex	Ethnicity	Phenotype	AOO	DD, y	Chronic cough	Cerebellar syndrome	Sensory neuropathy	Bilateral vestibular areflexia	Dysautonomia	Walking aid use (age, y)	Additional features
**AAGGC expansion**													
Case I-1	Allele 1: (AAGGG)_510_(AAGGC)_880_ Allele 2: (AAGGG)_940_(AAGGC)_900_	F	Caucasian (Indian)	CANVAS	24	17	Yes	Yes	Yes	Yes	No	Stick (36)	Cramps, pyramidal signs
Case I-2	Allele 1: (AAGGG)_n_(AAGGC)_n_ Allele 2: (AAGGG)_n_(AAGGC)_n_	F	Caucasian (Indian)	Sensory neuropathy + cough	34	8	Yes	N/A	Yes	N/A	N/A	No	–
**AGGGC expansion**													
Case II-1	Allele 1: (AGGGC)_1240_ Allele 2: (AAGGG)_930_	M	Mixed (Lebanese)	Sensory neuropathy + vestibular dysfunction	53	11	Yes	No	Yes	Yes	Yes	No	Cramps
Case III-1	Allele 1: (AGGGC)_3200_ Allele 2: (AAGGG)_1000_	M	Caucasian (British)	CANVAS	71	12	Yes	Yes	Yes	N/A	Yes	Wheelchair (81)	Cramps, cognitive/behavioural abnormalities after age 80
Case IV-1	Allele 1: (AGGGC)_1875_/ Allele 2: (AAGGG)_500_	M	Caucasian (Italian)	CANVAS	41	34	No	Yes	Yes	Yes	Yes	Wheelchair (72)	Cramps
Case V-1	Allele 1: (AGGGC)_n_/ Allele 2: (AAGGG)_n_	F	Caucasian (Italian)	Sensory neuropathy + cough	60	13	Yes	No	Yes	No	No	No	–
Case V-2	Allele 1: (AGGGC)_n_/ Allele 2: (AAGGG)_n_	F	Caucasian (Italian)	Sensory neuropathy	40	20	No	No	Yes	No	No	No	–
Case VI-1	Allele 1: (AGGGC)_n_/ Allele 2: (AAGGG)_n_	F	Caucasian (Italian)	Sensory ganglionopathy + cough	62	23	Yes	No	Yes	N/A	Yes	No	Voice and hand tremor, urinary incontinence
**AGAGG expansion**													
Case VII-1	Allele 1: (AAAGG)_470_(AGAGG)_470_/ Allele 2: (AAGGG)_1140_	F	Caucasian (British)	CANVAS	50	24	Yes	Yes	Yes	Yes	No	Walker (69), wheelchair (74)	–
**AAAGG expansion**													
Case VIII-1	Allele 1: (AAAGG)_610_(AAGGG)_390_/ Allele 2: (AAGGG)_1100_	M	Caucasian (British)	CANVAS	55	20	Yes	Yes	Yes	N/A	Yes	Walker and wheelchair (74)	Cognitive impairment since age 72
Case IX-1	Allele 1: (AAGGG)_700_(AAAGG)_200_/ Allele 2: (AAGGG)_1170_	M	Caucasian (British)	CANVAS	45	31	Yes	Yes	Yes	Yes	Yes	Walker (75)	RBD, positive DatScan
Case X-1	Allele 1: (AAAGG)_980_/ Allele 2: (AAGGG)_1010_	M	Caucasian (Australian)	CANVAS	58	15	Yes	Yes	Yes	Yes	N/A	N/A	–
Case XI-1	Allele 1: (AAAGG)_800_/ Allele 2: (AAGGG)_500_	F	Caucasian (Italian)	Sensory ganglionopathy + cough	73	3	Yes	No	Yes	No	No	Stick (77)	–
Case XII-1	Allele 1: (AAAGG)_600_/ Allele 2: (AAGGG)_390_	M	Caucasian (Italian)	Sensory ganglionopathy + cough	56	10	Yes	No	Yes	No	No	No	–

AOO = age of onset; CANVAS = cerebellar ataxia, neuropathy and vestibular areflexia syndrome; DD = disease duration; F = female; M = male; RBD = REM sleep behaviour disorder.

**Figure 2:**
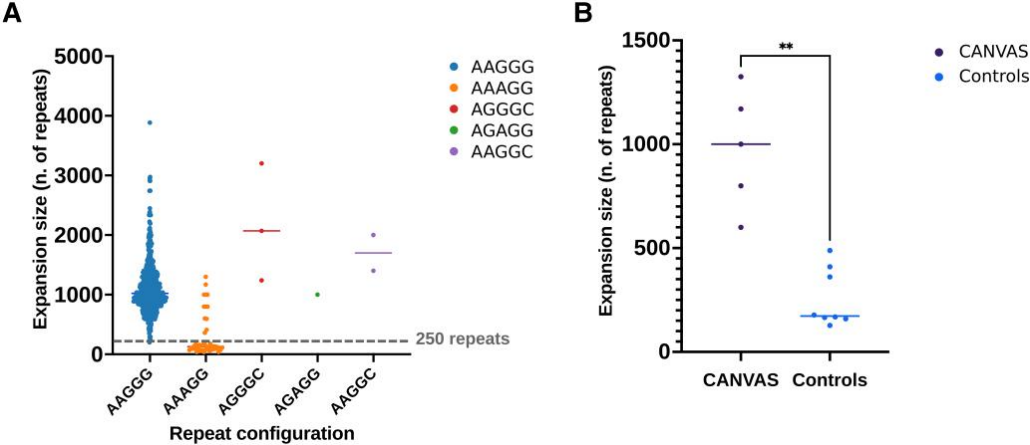
***RFC1* repeat expansion size.** (**A**) Comparison of repeat sizes of alleles carrying AAGGG, AAAGG, AAGGC, AGGGC and AGAGG expansions from this and previous studies.^1,5,6^ The dotted lines refer to the smallest pathogenic expansion of 250 AAGGG repeats identified so far. (**B**) Comparison of the AAAGG repeat sizes in the compound heterozygous state with the AAGGG expansion in patients with CANVAS/spectrum disorder versus controls. CANVAS = cerebellar ataxia, neuropathy and vestibular areflexia syndrome.

Based on Southern blotting, OGM or long-read sequencing (Fig. 1B and C) when available, we observed that the sizes of the rare AGGGC, AAGGC and AGAGG repeat expansions were >600 repeats in all cases [mean ± standard deviation (SD), 892 ± 247 repeat units] (Fig. 2A). Furthermore, enough DNA for Southern blotting was available from five patients with CANVAS/spectrum disorder (Cases VI–X), as defined by the presence of sensory neuropathy and at least one of the additional features of the full syndrome (cerebellar dysfunction, vestibular areflexia, cough), and eight controls carrying compound heterozygous AAGGG/AAAGG expansions (Fig. 2B).

In CANVAS patients, the AAAGG expansions were always ≥600 repeats (mean ± SD, 979 ± 257 repeat units) and were significantly larger than the AAAGG expansions (238 ± 142 repeat units) found in the controls (*P* < 0.0001), suggesting that, although the AAAGG repeat is usually small and non-pathogenic, as shown in Fig. 2A, larger AAAGG repeat expansions occur and may have a pathogenic role.

### Long-read sequencing confirms the sequence of the expanded repeats

To gain further insight into the exact sequence of the novel pathogenic motifs, we performed targeted long-read sequencing (Fig. 1D and Supplementary Table 1). We confirmed the presence of uninterrupted AGGGC_1240_ in Case II-1 and AGGGC_3200_ in Case III-1. Moreover, long-read sequencing enabled us to define the exact repeat composition of the AGAGG and AAGGC expansions, which revealed the presence of mixed repeat motifs (AAGGC)_900_(AAGGG)_940_ and (AGAGG)_470_(AAAGG)_470_ in Cases I-1 and VII-1, respectively. Long-read sequencing was also performed in five cases carrying large AAAGG expansions and showed the presence of uninterrupted AAAGG motifs in three (Cases X-1, XI-1 and XII-1), with sizes of 980, 800 and 600 repeat units, respectively, while two probands (Cases VIII-1 and IX-1) carried complex (AAAGG)_610_ (AAGGG)_390_ and (AAAGG)_700_(AAGGG)_200_ repeats.

### All pathogenic repeat configurations share an ancestral haplotype

**Figure 3:**
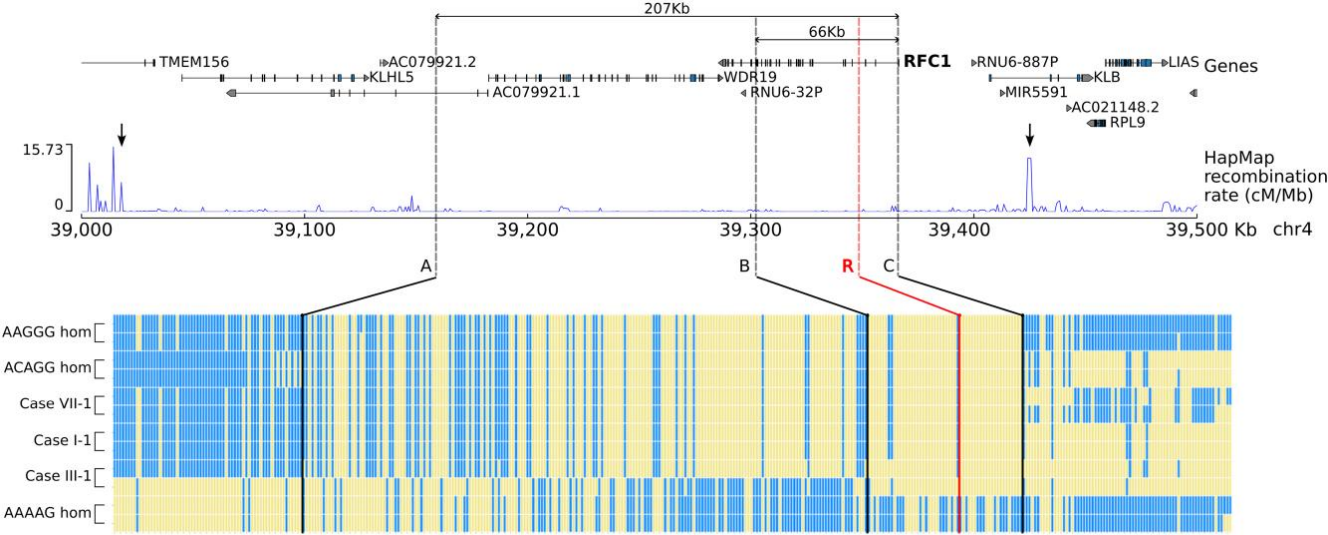
**A shared ancestral haplotype in patients with pathogenic *RFC1* motifs.** Graphical representation of the haplotypes associated with AAGGG, ACAGG and novel pathogenic repeat motifs identified in this study. For each single nucleotide polymorphism, the reference allele is represented in blue, while the alternative allele is represented in yellow. The repeat expansion locus is marked with a red line (R). There is a shared region (B–C, -rs2066782-rs6851075, chr4:39302305–39366034, hg38) of 66 kb for all novel configurations. A larger region of 207 kb (A–C, rs148316325- rs6851075, chr4:39158847–39366034, hg38), which is flanked by two recombination hotspots (arrows), is also shared among all but one allele for Case III-1, suggesting a recombination event at B (rs2066782) in this family. The shared haplotype lies in a region of low recombination rate (HapMap data) and is delimited by small peaks at A and C. A smaller increase in the recombination rate is also visible at B. hom = homozygous.

Subsequently, we looked at the inferred haplotypes associated with the novel pathogenic repeat motifs. A region of 66 kb (Fig. 3, between Markers B and C, chr4:39302305–39366034, hg38) was shared among all pathogenic alleles. It is worth noting that a larger region of 207 kb (between Markers A and C) containing the *WDR19* and *RFC1* genes was shared among all the pathogenic alleles, except one (Case III-1), where the haplotype became the same as the wild-type allele. This suggested a more recent recombination event at Marker B in Case III-1. The larger shared region identified in carriers of the novel pathogenic configurations, as well as in AAGGG and AAAGG carriers, supports the existence of an ancestral haplotype that gave rise to these expanded alleles. Notably, non-pathogenic AAAAG_(9–11)_ and expanded AAAAG repeats originated from a different haplotype.

We estimated that the ancestral haplotype that gave rise to different pathogenic repeat configurations in *RFC1* likely dates to 56 100 years ago (95% confidence interval: 27 680–115 580 years).

### Clinical features of patients carrying novel pathogenic repeat configurations in *RFC1*

We found 14 patients from 12 families carrying novel pathogenic *RFC1* repeat configurations. The demographic and clinical characteristics of patients are summarized in Table 2. All patients were Europeans, apart from Cases I-1 and I-2, who were from India, and Case X-1, who was from Australia. The mean age-of-onset was 51.5 ± 13.7 (24–73) years, and mean disease duration at examination was 17.2 years ± 8.7 (3–34) years. Six patients had isolated sensory neuropathy, which was associated with cough in four of them; one patient had sensory neuropathy and vestibular dysfunction; while seven cases had full CANVAS. Additional features were observed in some cases, including early onset and rapid progression (Case I-1), cognitive impairment (Cases III-1 and VI-1), muscle cramps (Cases I-1, II-1, III-1 and IV-1) and REM sleep behaviour disorder with positive dopamine transporter scan (DatScan) (Case IX-1). Autonomic dysfunction was observed in six cases, and in two of them (Cases II-1 and III-1), who both carried AGGGC expansions, it was severe and led to syncopal episodes. Detailed descriptions of the clinical features are provided in the Supplementary material.

### Pathogenic configurations in *RFC1* are predicted to form G-quadruplexes

As repetitive G-rich sequences are known to form G4s,^32,35,36^ secondary DNA structures which act as transcriptional regulators by impeding transcription factor binding to duplex-DNA or stalling the progression of RNA polymerase, we set out to evaluate the propensity of the different repeat configurations in *RFC1* to form G4s.

All pathogenic repeat configurations showed high G4 scores, which were in the range observed for the well-known G4-forming regions of the *cMYC*^37^ and *HRAS1*^38^ genes, as predicted by QGRS-Mapper and G4Hunter, in contrast to the non-pathogenic AAAAG (Table 3).

**Table 3:** Pathogenic *RFC1* motifs are predicted to form G-quadruplexes

Gene: analysed sequences	QGRS-Mapper score	G4Hunter score
*RFC1*: (AGGGC)_10_	42	1.83
*RFC1*: (AAGGG)_10_	42	2.00
*RFC1*: (AAGGC)_10_	21	1.82
*RFC1*: (AAAGG)_10_	21	0.94
*RFC1*: (AGAGG)_10_	21	1.12
*RFC1:* (AAAAG)_10_	No putative G4 identified	
*c-MYC*: TGGGGAGGTGGGGAGGGTGGGGAAGG	41	2.59
*HRAS-1*: TCGGGTTGCGGCGCAGGCACGGGCG	41	1.19

G-score values comparison between repeat configurations found in *RFC1* and well-known G4-forming sequences.

## Discussion

**Figure 4:**
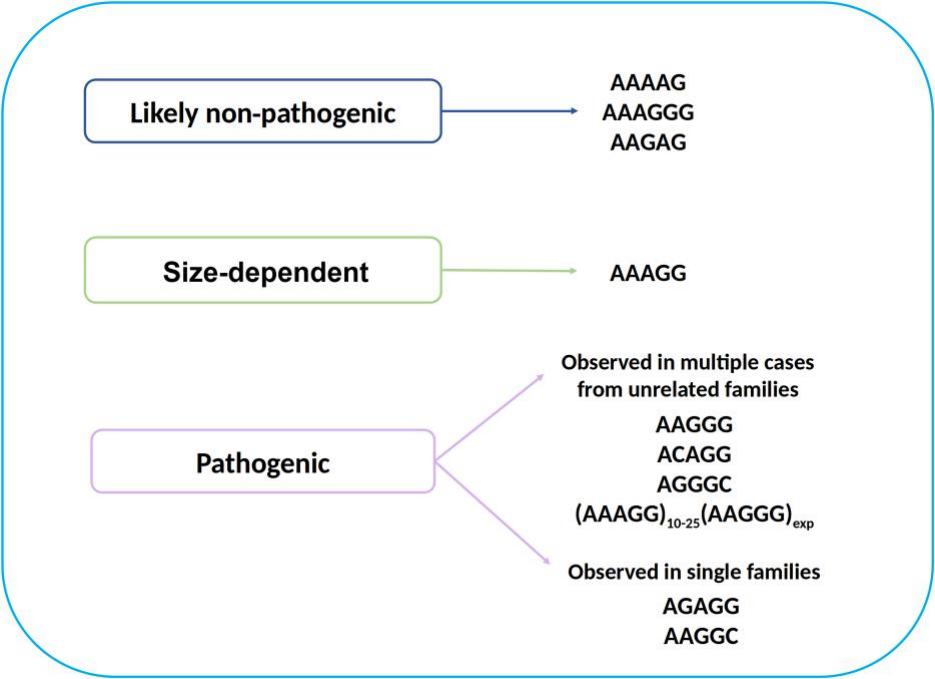
Normal and pathogenic significance of repeat expansion motifs at the *RFC1* locus.

We leveraged WGS data from nearly 10 000 individuals recruited to the Genomics England sequencing project to investigate the normal and pathogenic variation of the *RFC1* repeat. We identified three novel repeat configurations associated with CANVAS/spectrum disorder, including AGGGC, AAGGC and AGAGG. Notably, we also showed a pathogenic role for large uninterrupted or interrupted AAAGG expansions, whereas AAAAG, AAGAG and AAAGGG expansions are likely always to be benign (Fig. 4).

Most pathogenic repeat expansions were found in individuals of Caucasian ancestry; however, ACAGG seemed to be common in East Asians, while AAGGC was identified in a family of South Asian ancestry. Interestingly, most pathogenic repeats seem to have arisen from a shared region of 207 kb, supporting their origin from a common ancestor who lived ∼50 000 years ago. Rafehi *et al*.^2^ previously identified a larger ancestral haplotype in Australian patients affected by CANVAS of 360 kb and estimated that the most recent common ancestor lived approximately 25 880 (confidence interval: 14 080–48 020) years ago.^2^ In our study, the inclusion of additional pathogenic repeat configurations and multiple ethnicities allowed the identification of a smaller core haplotype and has extended further back in time the origin of the common ancestor carrying a pathogenic repeat in *RFC1*. It is reasonable to believe that the occurrence of subsequent A–G transitions and A–G or G-C transversions in the poly-A tail of the AluxSx3 element on the ancestral haplotype favoured the further expansion of GC-rich motifs over the millennia. Since the most significant recent wave out of Africa is estimated to have taken place about 70 000–50 000 years ago, we can speculate that the repeat-containing haplotype spread with the migration of early modern humans from Africa through the Near East and to the rest of the world.

Patients showed clinical features undistinguishable from those of patients carrying biallelic AAGGG expansions. In some cases, however, the disease appeared to be more severe due to symptomatic dysautonomia, early cerebellar involvement or disabling gait disturbance.

The identification of these motifs has direct clinical implications. Given their frequency, RP-PCR for AAAGG and AGGGC should be considered in all cases. Particular attention should be paid to carriers of compound AAGGG/AAAGG expansions and accurate sizing, and full sequencing of the satellite through long-read sequencing is recommended to establish its possible pathogenicity. In addition, depending on availability, Southern blotting, genome optical mapping or long-read sequencing are warranted in patients with a suggestive clinical phenotype but inconclusive screening, such as in cases with absence of a PCR-amplifiable product on flanking PCR but negative RP-PCR for AAGGG expansion.

The findings of this study highlight the genetic complexity of *RFC1*-related disease and lend support to the hypothesis that the size and GC-content of the pathogenic repeat is more important than the exact repeat motif. Consistently, all pathogenic repeat configurations are rich in G-content and are predicted to form highly stable G4s, which have previously been demonstrated to affect gene transcription in other pathogenic conditions.^35,36^

Both Nanopore or PacBio sequencing platforms and either the targeted CRISPR/Cas9 or adaptive selection approach were used to increase the accuracy of the sequencing of the *RFC1* repeat locus. Despite several attempts and similarly to other large satellites, long-read sequencing of the *RFC1* repeat remained challenging and, depending on the specific configurations, size and DNA quality, only a few reads were available for analysis in some cases. Notably, uneven coverage at the *RFC1* locus across samples was also observed in a recent study of *RFC1* repeat composition using Nanopore sequencing.^19^ The authors attributed the variability to variable degrees of DNA fragmentation depending on the delay between blood sampling and DNA extraction. Hopefully, constant advancements in long-read sequencing platforms and a decrease in cost (currently ∼US$1000 per sample) will soon translate into increased accessibility to this technology and higher levels of accuracy.

In conclusion, this study expanded the genetic heterogeneity underlying *RFC1* CANVAS/spectrum disorder and identified three additional pathogenic AAGGC, AGGGC and AGAGG repeat motifs. We also demonstrated a pathogenic role for large uninterrupted or interrupted AAAGG expansions, thereby highlighting the importance of sizing and, if possible, full sequencing of the *RFC1* satellite expansion in clinically selected cases, to correctly diagnose and counsel patients and their families.

## Supplementary Material

awad240_Supplementary_Data

## Data Availability

Anonymized data are available from the corresponding author.

## Appendix 1

### Genomics England Research Consortium

J. C. Ambrose, P. Arumugam, E. L. Baple, M. Bleda, F. Boardman-Pretty, J. M. Boissiere, C. R. Boustred, H. Brittain, M. J. Caulfield, G. C. Chan, C. E. H. Craig, L. C. Daugherty, A. de Burca, A. Devereau, G. Elgar, R. E. Foulger, T. Fowler, P. Furió-Tarí, E. Gustavsson, J. M. Hackett, D. Halai, A. Hamblin, S. Henderson, J. E. Holman, T. J. P. Hubbard, K. Ibáñez, R. Jackson, L. J. Jones, D. Kasperaviciute, M. Kayikci, L. Lahnstein, K. Lawson, S. E. A. Leigh, I. U. S. Leong, F. J. Lopez, F. Maleady-Crowe, J. Mason, E. M. McDonagh, L. Moutsianas, M. Mueller, N. Murugaesu, A. C. Need, C. A. Odhams, C. Patch, D. Perez-Gil, D. Polychronopoulos, J. Pullinger, T. Rahim, A. Rendon, P. Riesgo-Ferreiro, T. Rogers, M. Ryten, B. Rugginini, K. Savage, K. Sawant, R. H. Scott, A. Siddiq, A. Sieghart, D. Smedley, K. R. Smith, A. Sosinsky, W. Spooner, H. E. Stevens, A. Stuckey, R. Sultana, E. R. A. Thomas, S. R. Thompson, C. Tregidgo, A. Tucci, E. Walsh, S. A. Watters, M. J. Welland, E. Williams, K. Witkowska, S. M. Wood, M. Zarowiecki. Further details are available in the Supplementary material.
