# Correction to: Normal and pathogenic variation of RFC1 repeat expansions: implications for clinical diagnosis

**DOI:** 10.1093/brain/awad386

**Published:** 2023-11-16

Natalia Dominik, Stefania Magri, Riccardo Currò, Elena Abati, Stefano Facchini, Marinella Corbetta, Hannah Macpherson, Daniela Di Bella, Elisa Sarto, Igor Stevanovski, Sanjog R. Chintalaphani, Fulya Akcimen, Arianna Manini, Elisa Vegezzi, Ilaria Quartesan, Kylie-Ann Montgomery, Valentina Pirota, Emmanuele Crespan, Cecilia Perini, Glenda Paola Grupelli, Pedro J. Tomaselli, Wilson Marques, Genomics England Research Consortium, Joseph Shaw, James Polke, Ettore Salsano, Silvia Fenu, Davide Pareyson, Chiara Pisciotta, George K. Tofaris, Andrea H. Nemeth, John Ealing, Aleksandar Radunovic, Seamus Kearney, Kishore R. Kumar, Steve Vucic, Marina Kennerson, Mary M. Reilly, Henry Houlden, Ira Deveson, Arianna Tucci, Franco Taroni, Andrea Cortese. Normal and pathogenic variation of *RFC1* repeat expansions: implications for clinical diagnosis. *Brain*. 2023;146:5060–5069. https://doi.org/10.1093/brain/awad240

In the original article, the affiliations for the author Valentina Pirota incorrectly included the Institute of Molecular Genetics IGM-CNR ‘Luigi Luca Cavalli-Sforza’, Pavia 27100, Italy.

This has been corrected and the affiliations updated.

