# Supplementary material for: Normal and pathogenic variation of RFC1 repeat expansions: implications for clinical diagnosis
Source: Brain. 2023 Jul 14;146(12):5060–9. doi: 10.1093/brain/awad240 (PMC10689911; doi:10.1093/brain/awad240)
Supplement: awad240_Supplementary_Data [file awad240_supplementary_data.zip › brain-2023-00600-File009.pdf]

Supplementary material

Supplementary Table 1. Long read sequencing of *RFC1* repeat expansion

		Number of reads		
**Case ID**	**Repeat motifs**	**Oxford Nanopore (CRISPR/Cas9 enrichment)**	**Oxford Nano pore (adaptive selection)**	**Pacific Biosciences (CRISPR/Cas9 enrichment)**
**Case I-1**	Allele 1: $(AAGGG)_{510}$ $(AAGGC)_{880}$<br>Allele 2: $(AAGGG)_{940}$ $(AAGGC)_{900}$	2 complete reads;<br>10 partial reads	9 complete reads	35 complete reads
**Case I-2**	Allele 1: $(AAGGG)_n(AAGGC)_n$<br>Allele 2: $(AAGGG)_n(AAGGC)_n$	Not performed	Not performed	Not performed
**Case II-1**	Allele 1: $(AGGGC)_{1240}$<br>Allele 2: $(AAGGG)_{930}$	Not performed	7 complete reads	3 complete reads
**Case III-1**	Allele 1: $(AGGGC)_{3200}$<br>Allele 2: $(AAGGG)_{1000}$	20 complete reads;<br>13 partial reads	Not performed	4 complete reads;<br>4 partial reads
**Case IV-1**	Allele 1: $(AGGGC)_{1875}$<br>Allele 2: $(AAGGG)_{500}$	7 complete reads;<br>22 partial reads	Not performed	Not performed
**Case V-1**	Allele 1: $(AGGGC)_n$<br>Allele 2: $(AAGGG)_n$	Not performed	Not performed	Not performed
**Case V-2**	Allele 1: $(AGGGC)_n$<br>Allele 2: $(AAGGG)_n$	Not performed	Not performed	Not performed
**Case VI-1**	Allele 1: $(AGGGC)_n$/<br>Allele 2: $(AAGGG)_n$	Not performed	Not performed	Not performed
**Case VII-1**	Allele 1: $(AAAGG)_{470}(AGAGG)_{470}$<br>Allele 2: $(AAGGG)_{1140}$	2 complete reads;<br>12 partial reads	Not performed	12 complete reads;<br>3 complete reads
**Case VIII-1**	Allele 1: $(AAAGG)_{610}(AAGGG)_{390}$<br>Allele 2: $(AAGGG)_{1100}$	Not performed	2 complete reads	Not performed
**Case IX-1**	Allele 1: $(AAGGG)_{700}(AAAGG)_{200}$<br>Allele 2: $(AAGGG)_{1170}$	4 partial reads	4 complete reads;<br>5 partial reads	1 complete reads;<br>1 partial reads
**Case X-1**	Allele 1: $(AAAGG)_{980}$<br>Allele 2: $(AAGGG)_{1010}$	Not performed	22 complete reads	Not performed
**Case XI-1**	Allele 1: $(AAAGG)_{800}$<br>Allele 2: $(AAGGG)_{500}$	25 complete reads;<br>87 partial reads	Not performed	Not performed
**Case XII-1**	Allele 1: $(AAAGG)_{600}$<br>Allele 2: $(AAGGG)_{390}$	2 complete reads;<br>8 partial reads	Not performed	Not performed

Partial reads are defines as reads which did not span the entire RFC1 repeat expansion locus.

Supplementary Table 2. Ethnicity distribution

A. Controls

		European	South Asian	African	East Asian	American	Mixed	Total
**Biallelic with AAGGG**	AAAAG	224	4	0	0	0	20	248
	AAAGGG	26	3	0	0	0	3	32
	AAGAG	14	1	0	0	0	1	16
	AAAGG	43	2	0	0	0	2	47
	ACGGG	0	0	0	0	0	0	0
	AGAGG	0	0	0	0	0	0	0
	AGGGC	0	0	0	0	0	0	0
**Homozygous**	ACAGG	0	0	0	0	0	0	0
	AAGGC	0	0	0	0	0	0	0
**Overall**		6214	802	165	43	17	866	8107

B. Ataxia cases

		European	South Asian	African	East Asian	American	Mixed	Total
**Biallelic with AAGGG**	AAAAG	20	0	0	0	0	1	21
	AAAGGG	4	0	0	0	0	1	5
	AAGAG	3	0	0	0	0	0	3
	AAAGG	8	0	0	0	0	2	10
	ACGGG	1	0	0	0	0	0	1
	AGAGG	1	0	0	0	0	0	1
	AGGGC	0	0	0	0	0	1	1
**Homozygous**	ACAGG	0	0	0	1	0	0	1
	AAGGC	0	1	0	0	0	0	1
**Overall**		740	59	11	3	0	80	893

Supplementary note. Clinical description of cases carrying novel pathogenic motifs in *RFC1*

Case I-1, who carries the homozygous (AAGGC)(AAGGG) repeat, showed early onset and rapid progression. Neurological exam revealed prominent cerebellar involvement and bilateral vestibular dysfunction. Notably, despite the presence of a severe sensory neuropathy at neurophysiological testing, the patient reported only mild clinical sensory involvement, with reduced pinprick sensation with preserved vibration and joint position sense. In contrast with

the classic CANVAS phenotype, she also showed pyramidal signs such as brisk reflexes and mild spasticity in the lower limbs. Interestingly, she had severe cerebellar atrophy particularly involving the vermian lobules and, in a milder fashion, also cerebellar hemispheres, atrophy of the middle cerebellar peduncles and thinning of cervical spinal cord at Magnetic Resonance Imaging (MRI) scans.

She also has one older sister (proband I-2), who complains of mild unsteadiness since the age of 34 and chronic cough since the age of 25. She underwent nerve conduction studies (NCS) showing the presence of sensory neuropathy. Proband I-2 lives abroad and could not be reviewed in person; however a telephone consultation was conducted and DNA was collected. For this reason, cerebellar and autonomic functions could not be assessed.

Probands II-1, III-1, IV-1, V-1, V-2 and VI-1 carry an AGGGC expansion in compound heterozygosis with the canonical AAGGG repeat. Two cases display classic CANVAS phenotype. Proband III-1 showcased a late onset with fast progression, and was wheelchair-bound after a 10-years disease duration. Additionally, he developed signs of cognitive impairment, with personality changes and impulsive behaviours. Proband IV-1 experienced its first symptoms at the age of 41, but his progression was slower, and he lost autonomous ambulation at the age of 71. Proband II-1 has a longstanding history of sensory axonal neuropathy and vestibular dysfunction, but never developed cerebellar involvement. Cases V-1 and V-2 are siblings and they display sensory neuropathy with cough. Case VI-1 is affected by sensory neuropathy alongside with chronic cough and voice and hand tremor.

In addition to these features, four patients (II-1, III-1, IV-1 and VI-1) report autonomic dysfunction. Proband II-1 and proband III-1 display a severe dysautonomia, resulting in syncopal episodes, excessive sweating and ocular dryness in the case of proband II-1 and in erectile dysfunction and frequent syncopal episodes in the case of proband III-2. Proband IV-1 showcased a more benign dysautonomia, with mild cardiac and vascular parasympathetic alteration. Proband VI-1 presents urinary incontinence. It is also worth noting that three patients (II-1, III-1 and IV-1) also suffer from muscle cramps at the level of the thighs. Interestingly, NCS studies conducted on proband II-1 revealed fasciculations and denervation in the distal upper and lower limb muscles sampled.

Proband VII-1, who carries the compound heterozygous expansion (AAGGG) / (AGAGG) (AAAGG), reports symptom onset at the age of 45. She presents prominent cerebellar features and a rather mild sensory involvement, resulting in an initial diagnosis as MSA. However, she

did not develop dysautonomia nor parkinsonism after a disease duration of 24 years. DatScan was performed, yielding normal results. Moreover, she presents a chronic cough and bilaterally altered VOR. She has been using a walker since the age of 68 and wheelchair since the age of 74.

Cases VIII-1, IX-1, X-1, XI-1 and XII-1 all carry the compound heterozygous expansion AAAGG, either pure or as part of complex motifs with AAGGG interruptions. Cases VIII-1 and IX-1, who carry an interrupted AAAGG motif, present a full CANVAS phenotype with onset in the fifth or sixth decade. They also report dysautonomic symptoms (urinary dysfunction and constipation in proband VIII-1, erectile dysfunction in proband IX-1). Both were wheelchair-bound by the age of 75. Additionally, proband VIII-1 experienced a cognitive decline since the age of 72, with his wife reporting increased difficulties with memory for recent events. Neuropsychological testing revealed an impairment in the non-verbal domain and in attentional and executive functioning, therefore suggesting a degree of anterior compromise. Conversely, proband IX-1 has a history of REM sleep behavior disorder, for which he performed a dopamine transporter scan (DatScan) that interestingly showed truncation of the left putamen and diminished uptake in the right caudate and the putamen. No other extrapyramidal features were reported nor detected. His family also reports a recent onset of memory disturbances, particularly for recent events, which was however not investigated further.

Cases X-1, XI-1 and XII-1 were found to carry pure AAAGG expansions. Repeat size of case X-1 is 1000 repeats and he presents full CANVAS disease, with age of onset of 58. This patient has already been reported in *Stevanovski I et al., 2022*. Conversely, cases XI-1 and XII-1 were found to have AAAGG expansions with sizes below 800 repeats. They are affected by a longstanding sensory ganglionopathy combined with chronic cough, with age of onset of 73 and 56 respectively. No symptom nor sign of dysautonomia was observed.
