# Supplementary material for: Normal and pathogenic variation of RFC1 repeat expansions: implications for clinical diagnosis
Source: Brain. 2023 Jul 14;146(12):5060–9. doi: 10.1093/brain/awad240 (PMC10689911; doi:10.1093/brain/awad240)
Supplement: awad240_Supplementary_Data [file awad240_supplementary_data.zip › brain-2023-00600-File010.pdf]

Appendix 1

Genomics England Research Consortium: list of collaborators

The members of The Genomics England Research Consortium are:

J. C. Ambrose[1], P. Arumugam[1], E. L. Baple[1], M. Bleda[1], F. Boardman-Pretty[1,2], J. M. Boissiere[1], C. R. Boustred[1], H. Brittain[1], M. J. Caulfield[1,2], G. C. Chan[1], C. E. H. Craig[1], L. C. Daugherty[1], A. de Burca[1], A. Devereau[1], G. Elgar[1,2], R. E. Foulger[1], T. Fowler[1], P. Furió-Tarí[1], E. Gustavsson, J. M. Hackett[1], D. Halai[1], A. Hamblin[1], S. Henderson[1,2], J. E. Holman[1], T. J. P. Hubbard[1], K. Ibáñez[1,2], R. Jackson[1], L. J. Jones[1,2], D. Kasperaviciute[1,2], M. Kayikci[1], L. Lahnstein[1], K. Lawson[1], S. E. A. Leigh[1], I. U. S. Leong[1], F. J. Lopez[1], F. Maleady-Crowe[1], J. Mason[1], E. M. McDonagh[1,2], L. Moutsianas[1,2], M. Mueller[1,2], N. Murugaesu[1], A. C. Need[1,2], C. A. Odhams[1], C. Patch[1,2], D. Perez-Gil[1], D. Polychronopoulos[1], J. Pullinger[1], T. Rahim[1], A. Rendon[1], P. Riesgo-Ferreiro[1], T. Rogers[1], M. Ryten[1], B. Rugginini, K. Savage[1], K. Sawant[1], R. H. Scott[1], A. Siddiq[1], A. Sieghart[1], D. Smedley[1,2], K. R. Smith[1,2], A. Sosinsky[1,2], W. Spooner[1], H. E. Stevens[1], A. Stuckey[1], R. Sultana[1], E. R. A. Thomas[1,2], S. R. Thompson[1], C. Tregidgo[1], A. Tucci[1,2], E. Walsh[1], S. A. Watters[1], M. J. Welland[1], E. Williams[1], K. Witkowska[1,2], S. M. Wood[1,2], M. Zarowiecki[1]

1. Genomics England, London, UK
2. William Harvey Research Institute, Queen Mary University of London, London, EC1M 6BQ, UK
